# Correction

**DOI:** 10.1080/15592294.2024.2388007

**Published:** 2024-08-04

**Article title**: Hypermethylation of PI3K-AKT signalling pathway genes is associated with human neural tube defects

**Authors**: Tian Tian, Xinyuan Lai, Kuanhui Xiang, Xiao Han, Shengju Yin, Robert M. Cabrera, John W. Steele, Yunping Lei, Xuanye Cao, Richard H. Finnell, Linlin Wang, and Aiguo Ren

**Journal**: *Epigenetics*

**Bibliometrics**: Volume 17, Number 02, pages 133-146

**DOI**: https://doi.org/10.1080/15592294.2021.1878725

The author provided updated version of Declaration Statement after publication. The corrected Declaration Statement has been placed below. This correction has not changed the description, interpretation, or the original conclusions of the article. The authors apologize for any inconvenience caused.

**Declaration Statement:**

Dr. Finnell and Cabrera formerly held a leadership position with the now dissolved TeratOmic Consulting LLC. Dr. Cabrera currently heads DARTox Consulting, LLC. Dr. Finnell also receives travel funds to attend editorial board meetings of the Journal of Reproductive and Developmental Medicine published out of the Red Hospital of Fudan University.

